# Intranasal booster using an Omicron vaccine confers broad mucosal and systemic immunity against SARS-CoV-2 variants

**DOI:** 10.1038/s41392-023-01423-6

**Published:** 2023-04-17

**Authors:** Qian Wang, Chenchen Yang, Li Yin, Jing Sun, Wei Wang, Hengchun Li, Zhengyuan Zhang, Si Chen, Bo Liu, Zijian Liu, Linjing Shi, Xiaolin Liu, Suhua Guan, Chunhua Wang, Linbing Qu, Ying Feng, Xuefeng Niu, Liqiang Feng, Jincun Zhao, Pingchao Li, Ling Chen, Nanshan Zhong

**Affiliations:** 1 State Key Laboratory of Respiratory Disease, Guangzhou Institute of Respiratory Health, The First Affiliated Hospital of Guangzhou Medical University, Guangzhou, China; 2 Guangzhou nBiomed Ltd., Guangzhou, China; 3 Guangzhou Laboratory & Bioland Laboratory, Guangzhou, China; 4 Guangdong Laboratory of Computational Biomedicine, Guangzhou Institutes of Biomedicine and Health, Chinese Academy of Sciences, Guangzhou, China; 5 University of Chinese Academy of Sciences, Beijing, China

**Keywords:** Vaccines, Adaptive immunity

## Abstract

The highly contagious SARS-CoV-2 Omicron subvariants severely attenuated the effectiveness of currently licensed SARS-CoV-2 vaccines based on ancestral strains administered via intramuscular injection. In this study, we generated a recombinant, replication-incompetent human adenovirus type 5, Ad5-S-Omicron, that expresses Omicron BA.1 spike. Intranasal, but not intramuscular vaccination, elicited spike-specific respiratory mucosal IgA and residential T cell immune responses, in addition to systemic neutralizing antibodies and T cell immune responses against most Omicron subvariants. We tested intranasal Ad5-S-Omicron as a heterologous booster in mice that previously received intramuscular injection of inactivated ancestral vaccine. In addition to inducing serum broadly neutralizing antibodies, there was a significant induction of respiratory mucosal IgA and neutralizing activities against Omicron subvariants BA.1, BA.2, BA.5, BA.2.75, BF.7 as well as pre-Omicron strains Wildtype, Beta, and Delta. Serum and mucosal neutralizing activities against recently emerged XBB, BQ.1, and BQ.1.1 could also be detected but were much lower. Nasal lavage fluids from intranasal vaccination contained multimeric IgA that can bind to at least 10 spike proteins, including Omicron subvariants and pre-Omicron strains, and possessed broadly neutralizing activities. Intranasal vaccination using Ad5-S-Omicron or instillation of intranasal vaccinee’s nasal lavage fluids in mouse nostrils protected mice against Omicron challenge. Taken together, intranasal Ad5-S-Omicron booster on the basis of ancestral vaccines can establish effective mucosal and systemic immunity against Omicron subvariants and multiple SARS-CoV-2 variants. This candidate vaccine warrants further development as a safe, effective, and user-friendly infection and transmission-blocking vaccine.

## Introduction

It has been over 3 years since the beginning of the COVID-19 pandemic that is caused by SARS-CoV-2, which is an enveloped single-stranded RNA virus. Vaccines are the most effective way to minimize infection and associated morbidity and mortality. The spike protein of SARS-CoV-2 is the principal target for antibody and vaccine countermeasures. SARS-CoV-2 enters and replicates in epithelial cells through the binding of spike with the cell-surface receptor angiotensin-converting enzyme 2 (ACE2). As of March 06, 2023, over two-thirds of the world’s population has received at least one dose of a COVID-19 vaccine, and 13.23 billion doses have been administered globally.^1^ Although infection or vaccine-induced neutralizing antibodies can inhibit the binding and infection of SARS-CoV-2, the virus mutates rapidly. An increasing list of lineages is designated variants of concern (VOCs) due to increased transmission and evasion of vaccine-induced immunity, including Beta, Delta, and Omicron subvariants.

Since the end of 2021, the dominant variants have become and remained thus far the Omicron subvariants, including BA.1, BA.2, BA.2.12.1, BA.2.75, BA.4, BA.5, BF.7, BQ.1, BQ.1.1, and XBB. These subvariants contain multiple mutations with the capability of strong immune escape and rapid transmission. The effectiveness of the 2-dose mRNA-1273 vaccine against Omicron infection was 30.4% between 14–90 days and declined to 0% by 180 days post-vaccination.^2^ Even with the 4^th^ dose of mRNA vaccine of ancestral strain, vaccine efficacy against symptomatic infection was 30% for BNT162b2 and 11% for mRNA-1273, and people had a high viral load in the nasopharyngeal tract that can be highly transmissible.^3^ The outcome of reduced vaccine efficacy against new variants and lack of mucosal immunity may provide conditions for further selection of highly resistant and transmissible variants in the upper airway. Thus, there is a need to establish an immune barrier that can provide front-line immunity to block infection and transmission of Omicron subvariants.

SARS-CoV-2 infection starts in the upper respiratory system, where the nasopharyngeal tract is at the forefront. To prevent viruses from attaching and replicating at the mucosal epithelium, effective mucosal immunity in the airway is critically important. Earlier studies have shown that mucosal booster vaccination with adenovirus-vectored ancestral vaccines after mRNA priming can induce systemic and respiratory mucosal immunity and confer protection against the challenges of ancestral SARS-CoV-2 in mice.^4,5^ The respiratory tract contains a rich environment of immune cells, including macrophages, dendritic cells, T cells, and B cells. Nasal-associated lymphoid tissue (NALT), which is a constitutive structure of the nasal immune system, is part of mucosa-associated lymphoid tissue of the upper respiratory tract. NALT plays an important role in inducing the respiratory mucosal immune response, including the generation of Th cells and IgA-secreting B cells, which are different from other lymphoid tissues.^6,7^ Respiratory infection or mucosal vaccination can stimulate both systemic and mucosal immunity, which may provide sterilizing immunity to block virus infection.^7^ Some studies on respiratory viruses such as respiratory syncytial virus (RSV) and influenza virus provide substantial evidence that mucosal immunity is key to the effective control of respiratory viruses.^8–10^ The Global COVID-19 Vaccination Strategy in a Changing World updated in July 2022 posted by the WHO mentioned the importance of mucosal immunity in reducing SARS-CoV-2 transmission, which can help prevent the emergence of new VOCs and their global disease waves and related health and economic consequences.^11^ However, almost all COVID-19 vaccines approved for human usage, including inactivated virus, lipid nanoparticle-encapsulated mRNA, protein subunit of spike or RBD, and adenovirus vectored vaccines, are administered via intramuscular injection. These vaccines can induce a systemic immune response that protects against severe disease and mortality but not a mucosal immune response and are insufficient in preventing upper respiratory infection and transmission, especially with the emergence of highly transmissible Omicron subvariants.^12^

Human adenovirus serotype 5 (Ad5) is a no disease-associated respiratory virus that the majority of people have been infected with without notification.^13^ Recombinant Ad5 with deletion of the E1 and E3 regions is replication-incompetent and can only be produced in a complementing cell line that provides E1 gene products in trans. Therefore, replication-incompetent Ad5 can serve as an ideal vector to deliver antigens to the upper respiratory tract to induce a mucosal immune response. It has been demonstrated in clinical trials that intranasal administration of Ad5-vectored vaccines is safe and well tolerated.^14,15^ We previously demonstrated that intranasal administration of an Ad5 carrying spike of the Wildtype strain, can induce systemic and respiratory mucosal immune responses in animals and confer sterilizing-like protection in rhesus macaques challenged with the ancestral strain.^16,17^ In this study, we constructed an Ad5-vectored vaccine delivering Omicron BA.1 spike (Ad5-S-Omicron). We evaluated intranasal vaccination for inducing respiratory mucosal antibodies and T cell immune responses in mice. Since a large population has been vaccinated with inactivated whole-virus vaccine, we further evaluated intranasal vaccination with Ad5-S-Omicron on the basis of inactivated ancestral vaccine in inducing mucosal and systemic immune responses against Omicron subvariants in mice and a proof-of-principle human study.

## Results

### Construction of a replication-incompetent recombinant adenovirus for delivering the Omicron spike antigen as a candidate vaccine

**Fig. 1:**
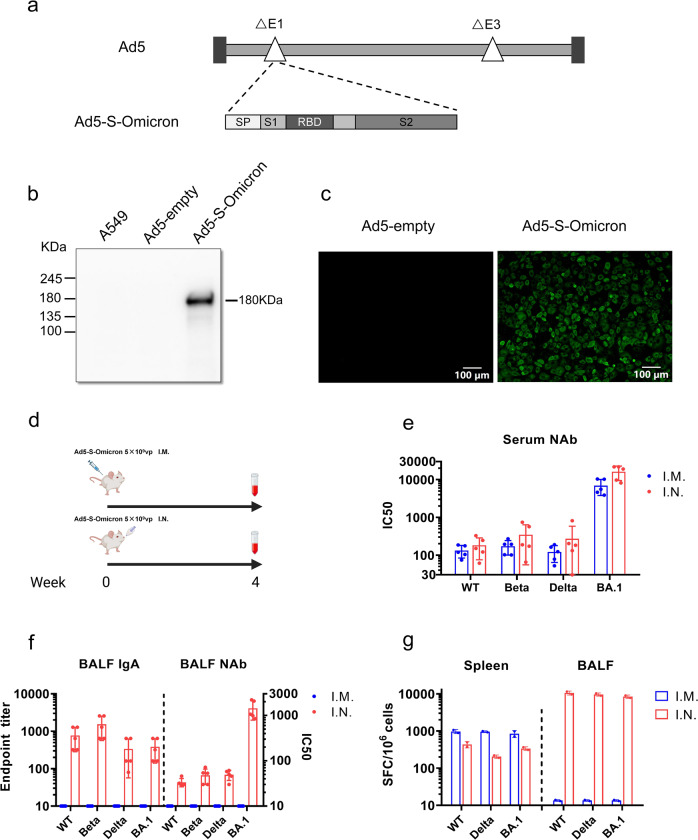
Construction of Ad5-S-Omicron and evaluation of immunogenicity of intranasal and intramuscular vaccination in mice. **a** Schematic diagram of the genome of Ad5-S-Omicron expressing spike protein of Omicron BA.1. **b** Western blot analysis of S protein expression in A549 cells infected with Ad5-S-Omicron at multiplicity of infection of 10. A549 cells infected with Ad5-empty were used as the negative controls. **c** Immunofluorescence analysis of S protein expression in A549 cells treated with Ad5-S-Omicron. A549 cells were infected with Ad5-S-Omicron or Ad5-empty at multiplicity of infection of 50. Twenty-four hours later, A549 cells were fixed and stained with an anti-spike antibody labeled with FITC. A549 cells were observed under fluorescence microscopy. Scale bar = 100 μm. **d** Schematic representation of the vaccination study regimen. Female 7-week-old BALB/c mice received a single vaccination of Ad5-S-Omicron (5 × 10^9^ vp, 1/10 of human dosage) either intranasally (I.N.) or intramuscularly (I.M.) (*n* = 5). At 4 weeks after vaccination, mice were sacrificed to collect serum, spleen, and BALF for analysis. **e** Pseudovirus neutralizing antibody (NAb) titers (IC50, 50% inhibitory concentration) against Wildtype, Beta, Delta, and Omicron BA.1 in serum samples. Data are shown as the means ± SD (*n* = 5), and individual data are presented. **f** Spike-specific IgA antibody endpoint titers in BALFs were measured by ELISA. Pseudovirus NAb titers in BALFs were measured as described earlier. Data are shown as the means ± SD, and individual data are presented. **g** IFN-γ ELISpot assay to measure antigen-specific T cell immune responses. Spleen or BALF cells from each group of 5 mice were pooled and incubated with spike peptide pools of Wildtype, Delta, and Omicron BA.1 for the detection of spike-specific IFN-γ-secreting cells in duplicate. Mice without vaccination showed no neutralizing titers over 1:10; thus, the data were not presented

We first generated a replication-incompetent recombinant adenovirus type 5, Ad5-S-Omicron, which can efficiently express the spike protein Omicron BA.1 in infected cells. The gene sequence (GISAID, accession No. EPI_ISL_6640919) encoding Omicron BA.1 spike protein (S-Omicron) was optimized by altering the codon usage, deleting the furin cleavage site, and replacing two prolines at amino acid positions 983 and 984 to enhance expression of spike protein in human cells. The expression cassette containing the S-Omicron gene driven by a human CMV promoter was inserted into the E1 region of E1- and E3-deleted Ad5 (Fig. 1a). Ad5-S-Omicron was rescued and propagated in human embryonic kidney (HEK) 293 cells. Infection of mammalian A549 cells with Ad5-S-Omicron confirmed the expression of Omicron BA.1 spike protein at the predicted molecular weight (Fig. 1b). Immunofluorescence staining revealed the expression of Omicron BA.1 spike protein on the cell surface (Fig. 1c).

### Ad5-S-Omicron administered intranasally but not intramuscularly elicits mucosal immunity, in addition to systemic immunity against Omicron subvariants

To assess the immunogenicity of Ad5-S-Omicron, we first compared intranasal and intramuscular vaccination in BALB/c mice at 5 × 10^9^ vp/dose, 1/10 of human dosage used by AstraZeneca, Johnson and Johnson, and CanSino for intramuscular injection of adenovirus vectored COVID-19 vaccines (Fig. 1d). Four weeks later, neutralizing antibody titers were measured using vesicular stomatitis virus (VSV) pseudotyped viruses bearing spike proteins of different SARS-CoV-2 strains.^18^ A single intranasal or intramuscular vaccination elicited robust serum neutralizing antibodies that skewed to BA.1, whereas the titers of neutralizing antibodies against pre-Omicron strains Wildtype, Beta, and Delta were at least 40-fold lower (Fig. 1e). We detected significant elevation of spike-specific IgA and neutralizing activity against BA.1 in bronchoalveolar lavage fluids (BALFs) from intranasally but not intramuscularly vaccinated mice (Fig. 1f). To assess T cell immune response, we detected the interferon (IFN)-γ-secreting cells in spleen and BALFs by enzyme-linked immune spot (ELISpot) using peptide pools of spike proteins Wildtype, Delta, and BA.1. Although intramuscular vaccination elicited higher systemic spike-specific T cell responses than intranasal vaccination, only intranasal, but not intramuscular vaccination, elicited spike-specific T cell response in the respiratory tract (Fig. 1g). Notably, the magnitude of spike-specific T cell response appeared to be comparable among Wildtype, Delta, and Omicron.

In addition, we compared Ad5-S-Omicron and Ad5-S-WT in BALB/c mice in a separate experiment to support the selection of Ad5-S-Omicron as an intranasal booster. A single intranasal injection of Ad5-S-Omicron elicited much higher neutralizing antibody titers against Omicron BA.1 and BA.2 than Ad5-S-WT. Ad5-S-WT elicited neutralizing antibodies skewed toward Wildtype, Beta, and Delta, with very low titers against Omicron BA.1 and BA.2 (Supplementary Fig. 1). Therefore, Ad5-S-Omicron but not Ad5-S-WT is a better candidate vaccine for inducing neutralizing antibodies against Omicron subvariants.

### Intranasal Ad5-S-Omicron booster on the basis of inactivated vaccine elicits mucosal and systemic neutralizing antibodies against both Omicron subvariants and pre-Omicron strains

**Fig. 2:**
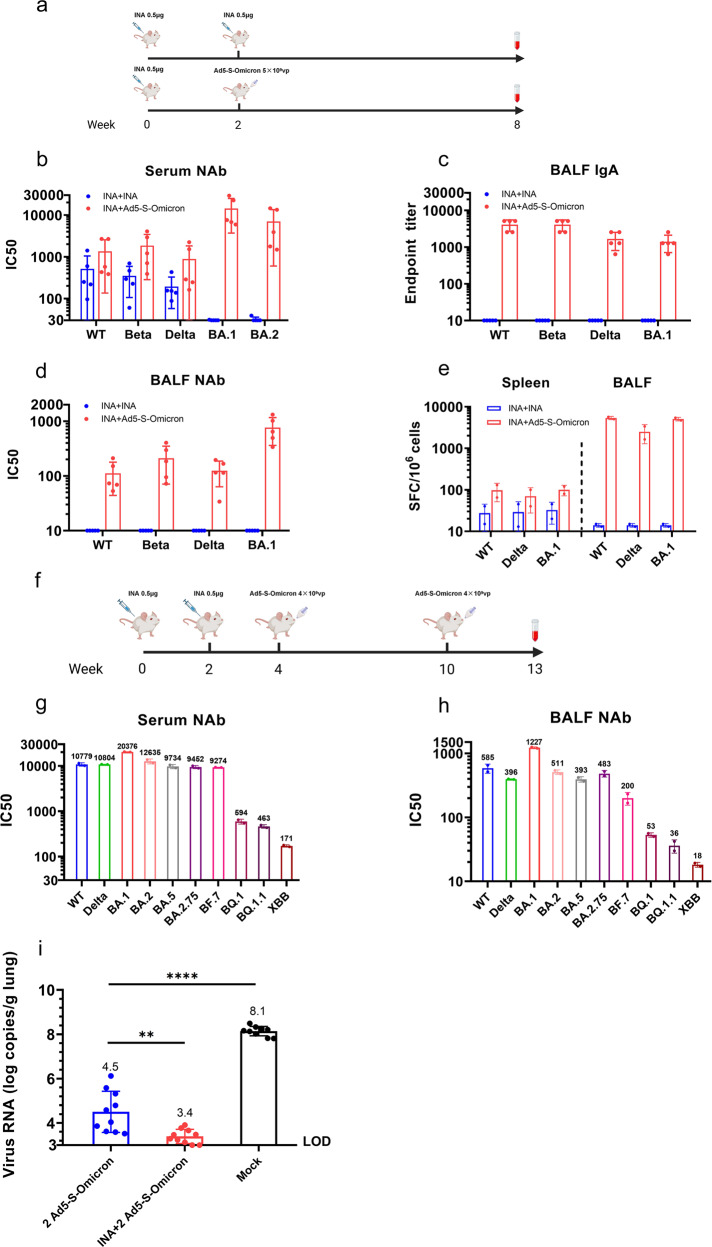
Intranasal Ad5-S-Omicron on the basis of inactivated vaccine establishes broad mucosal and systemic immunity and confers protection in mice. **a** Schematic diagram of immunization regimens. Female 7-week-old BALB/c mice were vaccinated with inactivated vaccine (INA) (0.5 μg, 1/10 of human dosage). Two weeks later, mice received either one homologous boost with 0.5 μg INA or one heterologous intranasal boost with Ad5-S-Omicron (5 × 10^9^ vp). At 6 weeks after the booster vaccination, serum, spleen, and BALF samples were collected for analysis. Serum (**b**) and BALF (**d**) pseudovirus NAb titers against Wildtype, Beta, Delta, Omicron BA.1, and BA.2. Data are shown as the means ± SD (*n* = 5). Individual data are presented. **c** Wildtype, Beta, Delta, and Omicron BA.1 spike-specific IgA antibody endpoint titers in BALFs. Data are shown as the means ± SD (*n* = 5), and individual data are presented. **e** IFN-γ ELISpot assay to measure the T cell immune response. Spleen or BALF cells from each group of 5 mice were isolated and pooled and incubated with spike peptide pools of Wildtype, Delta, and Omicron BA.1 for the detection of antigen-specific IFN-γ-secreting cells in duplicate. **f** Schematic diagram of vaccination regimens. Female 7-week-old BALB/c mice were vaccinated with 2 intramuscular injections of 0.5 μg inactivated vaccine (INA) and 2 intranasal boosts of 4 × 10^9^ vp Ad5-S-Omicron. Three weeks after the last booster vaccination, serum and BALF samples were collected for analysis. **g**–**h** Serum (**g**) or BALF (**h**) samples from each group of 5 mice were pooled in equal volume for the neutralization assay in duplicate. Pseudovirus neutralizing antibody titers against Wildtype, Delta, Omicron BA.1, BA.2, BA.5, BA2.75, BF.7, BQ.1, BQ.1.1, and XBB. **i** Female 7-week-old hACE2 transgenic mice were divided into 3 groups (10 mice per group): (1) two doses of intranasal Ad5-S-Omicron (4 × 10^9^ vp) at 2-week intervals; (2) two doses of intranasal Ad5-S-Omicron after vaccination with inactivated vaccine at 2-week intervals; and (3) mice without vaccination as control group. At 14 weeks after the initial vaccination, mice were challenged with intranasal instillation of 50,000 FFU Omicron BA.2.3. Three days later, mice were sacrificed and the lungs were collected for quantification of viral RNA using qPCR. Data are shown as the means ± SD (*n* = 10) and were analyzed with an unpaired *t* test (**P < 0.01, ****P < 0.0001). Individual data are presented. Mice without vaccination showed no neutralizing titers over 1:10; thus, the data were not presented

Because a large population has been vaccinated with inactivated ancestral vaccine, we next evaluated the effect of intranasal Ad5-Omicron as a heterologous booster on mucosal and systemic immunity (Fig. 2a). Intranasal Ad5-Omicron (5 × 10^9^ vp/dose) boost in BALB/c mice previously vaccinated with inactivated ancestral vaccine (0.5 μg, 1/10 of human dosage) elicited significantly higher serum neutralizing antibody titers against Omicron BA.1 (477.9-fold) and BA.2 (221.2-fold) compared to homologous boost using inactivated vaccine (Fig. 2b). Serum neutralizing titers were also elevated against pre-Omicron strains Wildtype (2.6-fold), Beta (5.4-fold), and Delta (4.6-fold) but to a much lesser extent than the increase against Omicron BA.1 and BA.2. A homologous boost of inactivated vaccine did not elicit significant level of neutralizing antibody titers against Omicron BA.1 and BA.2 and only modestly increased titers against pre-Omicron strains Wildtype, Beta, and Delta (Fig. 2b). Importantly, intranasal Ad5-S-Omicron elicited mucosal IgA against spike proteins of Wildtype, Beta, Delta, and Omicron BA.1 in BALFs (Fig. 2c). BALFs from these mice neutralized not only Omicron BA.1 but also Wildtype, Beta, and Delta to a lower level. In contrast, there were no detectable neutralizing activities in BALFs from mice that received 2 doses of inactivated vaccine (Fig. 2d). We also performed an IFN-γ ELISpot assay using spike peptide pools to evaluate the spike-specific T cell response. Intranasal Ad5-S-Omicron elicited not only systemic but also respiratory tract T cell responses against Wildtype, Delta, and Omicron BA.1. The magnitude of spike-specific T cell response in BALFs was 2 orders of magnitude higher than that of the splenic T cell response. In contrast, 2 doses of inactivated vaccine only elicited low level of spike-specific T cell response in the spleen (Fig. 2e).

We further evaluated the effect of two doses of intranasal Ad5-S-Omicron in BALB/c mice that previously received 2 doses of inactivated ancestral vaccine (Fig. 2f). We assessed the neutralization spectrum against a panel of 10 VSV pseudotyped viruses, including Omicron subvariants BA.1, BA.2, BA.5, BA.2.75, BF.7, BQ.1, BQ.1.1, and XBB, as well as pre-Omicron strains Wildtype and Delta. Serum neutralizing titers were higher against Omicron subvariants BA.1, BA.2, BA.5, BA.2.75, BF.7, Wildtype, and Delta. Neutralizing activities against most recently emerged BQ.1, BQ.1.1, and XBB were also detected but at a much lower level (Fig. 2g). Neutralizing activities in BALFs also showed similar spectrum as serum samples (Fig. 2h).

To understand whether intranasal booster vaccination with Ad5-S-Omicron on the basis of inactivated vaccine could confer protection in mice, we performed a challenge study using 10 hACE2 transgenic mice per group: (1) 2 doses of intranasal Ad5-S-Omicron; (2) 2 doses of intranasal Ad5-S-Omicron after vaccination with inactivated ancestral vaccine; and (3) no vaccination as control. Mice were challenged via intranasal instillation of Omicron BA.2.3 at 14 weeks after the initial vaccination. Three days after challenge, mice were sacrificed, and the lungs were collected for PCR quantification of the viral RNA. Compared to unvaccinated mice that had 8.1 logs of viral RNA copies/g lung, intranasal Ad5-S-Omicron boost after inactivated ancestral vaccine conferred 4.7 logs viral load reduction in the lungs. Of note, 2 doses of intranasal Ad5-S-Omicron also conferred a 3.6 log viral RNA reduction in the lungs (Fig. 2i).

Taken together, these results demonstrated that intranasal Ad5-S-Omicron can confer effective protection against Omicron infection. Furthermore, intranasal Ad5-S-Omicron on the basis of ancestral vaccine resulted in a broad spectrum of mucosal and systemic neutralizing activities against pre-Omicron strains Wildtype, Beta, and Delta, as well as Omicron subvariants BA.1, BA.2, BA.5, BA.2.75, BF.7, and low but detectable neutralizing activities against recently emerged BQ.1, BQ.1.1, and XBB.

### Intranasal vaccination-induced nasal IgA recognizes Omicron subvariants and pre-Omicron strains and can protect passively transferred mice from Omicron challenge

**Fig. 3:**
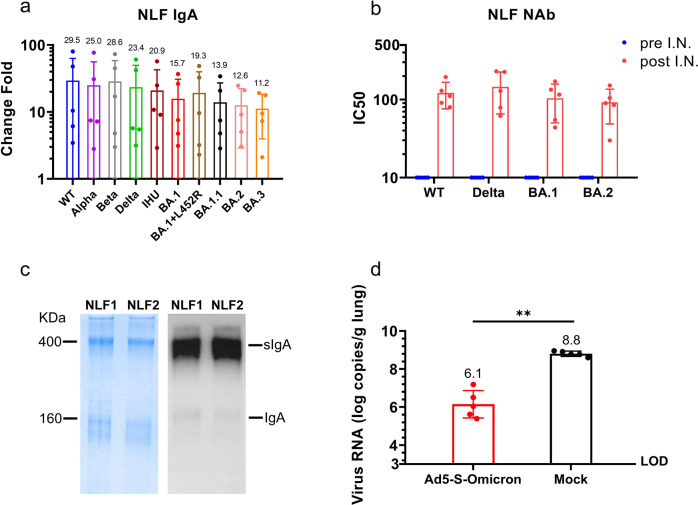
Detection of spike-specific secretory IgA and neutralizing activities in human nasal lavage fluids (NLFs). **a** Detection of secretory IgA that binds to spikes of Wildtype, Alpha (B.1.1.7), Beta (B.1.351), Delta (B.1.617.2), IHU (B.1.640.2), and Omicron BA.1, BA.2, BA.3, BA.1.1, and BA.1 + L452R in NLFs. NLFs were collected using a nasal irrigator with 40 ml saline before and 4 weeks after intranasal vaccination using 4 × 10^10^ vp Ad5-S-Omicron. IgA was measured using MSD panel 25 IgA kit (K15585U, Meso Scale Discovery, USA). A value above 3-fold higher than the baseline is considered seropositive. Data are shown as means ± SD (*n* = 5). **b** Pseudovirus neutralizing antibody titers against Wildtype, Delta, and Omicron BA.1 and BA.2 in concentrated nasal lavage fluid collected at day 0 and 4 weeks after intranasal spray of 4 × 10^10^ vp Ad5-S-Omicron. **c** Twenty microliters of NLFs from donors #1 (NLF1) and #2 (NLF2) were loaded onto SDS polyacrylamide gels for electrophoresis. Coomassie blue staining of the gel and Western blot analysis using anti-human IgA showed the dominant presence of dimeric IgA with a molecular weight of approximately 400 kDa. **d** Passive transfer of NLFs to mice conferred protection against the Omicron BA.1.1 challenge. One hundred microliters of concentrated NLFs from either donor #1 (NLF1) or one non-vaccinee were instilled into the nostrils of female 7-week-old BALB/c mice. Four hours later, mice were intranasally challenged with Omicron BA1.1 at 50,000 FFU. Three days later, mice were sacrificed and the lungs were collected for quantification of viral RNA by qPCR. Data are shown as the means ± SD (*n* = 5) and were analyzed with an unpaired *t* test (**P < 0.01). Individual data are presented

For proof of principle purposes, 5 co-authors of this report voluntarily took an intranasal spray of Ad5-S-Omicron that was prepared for clinical trial. These intranasal vaccinees have previously received two doses of inactivated ancestral vaccine over six months ago. Using Meso Scale Discovery (MSD) chemiluminescent method to detect IgA that binds to ten spike proteins, we found a significant induction of Omicron BA.1 (15.7-fold), BA.2 (12.6-fold), BA.3 (11.2-fold), BA.1.1 (13.9-fold), BA.1 + L452R/BA.5 like (19.3-fold), IHU/B.1.640.2 (20.9-fold), as well as Wildtype (29.5-fold), Beta (28.6-fold), and Delta (23.4-fold) over baseline levels in nasal lavage fluids (NLFs) collected at 4 weeks after intranasal vaccination (Fig. 3a). We could detect neutralizing activities against Wildtype, Delta, Omicron BA.1, and BA.2 in NLFs collected after intranasal vaccination (Fig. 3b). Coomassie blue staining of SDS-PAGE analysis and Western blot analysis using anti-IgA antibody revealed that dimeric or multimeric IgA is the dominant protein in NLFs (Fig. 3c). We subsequently carried out a passive transfer study by intranasally instilling 100 μl concentrated NLFs into mouse nostril and challenged these mice with Omicron BA.1.1. PCR quantification of the viral RNA in the lungs collected 3 days later revealed a 2.7-log viral load reduction in mice that received NLFs from an intranasal vaccinee. In contrast, there was a high viral load (8.8 log copies/g lung) in mice that received NLFs from a non-intranasal vaccinee (Fig. 3d). These results demonstrated that nasal secretory IgA from intranasal Ad5-S-Omicron vaccinees can neutralize Omicron subvariants and pre-Omicron strains, thus providing first-line defense against a wide spectrum of SARS-CoV-2 variants.

## Discussion

Omicron subvariants have become the dominant variants worldwide due to their increased transmissibility and immune escape capacity. Our study highlighted that intranasal boost with Ad5-S-Omicron after inactivated ancestral vaccine leads to significant induction of mucosal and systemic neutralizing antibodies against pre-Omicron strains Wildtype, Beta, Delta, and Omicron subvariants, including BA.1, BA.2, BA.5, BA.2.75, BF.7 and to a lesser extent BQ.1, BQ.1.1 and XBB in mice. Consistent with observations in an animal model with adenovirus vectored vaccine (Wildtype) booster,^5^ the intranasal booster with Ad5-S-Omicron induces respiratory mucosal spike-specific IgA in humans, which also has broad neutralizing activities, including Wildtype, Beta, Delta, and Omicron. We found that secretory dimeric IgA is the dominant protein in human NLFs collected from intranasal vaccinees, which can bind to 10 spike proteins of Omicron subvariants and pre-Omicron strains. Most intriguingly, transfer of NLFs into mouse nostrils conferred protection against Omicron challenge. Therefore, intranasal booster with Ad5-S-Omicron can elicit nasal secretory IgA that alone is highly potent in providing the front-line defense against Omicron infection.

Currently, almost all regulatory-approved COVID-19 vaccines are administered via intramuscular injection. Although most of these vaccines have been shown to reduce severe disease and mortality, they are not highly effective in completely blocking infection of Omicron subvariants, which evolve to preferentially infect and replicate in the upper respiratory pathway, especially the nose.^19^ Vaccine effectiveness also wanes rapidly, usually in less than 6 months.^12^ It has been reported that the best protection comes from people with “hybrid immunity”, i.e., who have both infection and vaccinations. The effectiveness against Omicron symptomatic infection increased from 52.2% in people with 3 doses of mRNA vaccines to 77.3% in people with “hybrid immunity”, i.e., 3 doses of mRNA vaccines plus an infection before the Omicron pandemic.^20^ A key difference between SARS-CoV-2 infection-induced immunity and intramuscular vaccination-induced immunity is that natural infection induces respiratory mucosal immunity. An important hallmark of mucosal immunity is the production of antigen-specific secretory IgA antibodies. Several studies have reported that spike-specific mucosal IgA in bronchoalveolar lavages, saliva, or nasal swabs can be detected in people who have recovered from SARS-CoV-2 infection.^21–23^ Unlike monomeric serum IgA, mucosal secretory IgA is produced locally and is present in dimeric and multimeric forms that can provide the first-line blockage against Omicron entry via upper respiratory tract.

We found that the inactivated ancestral vaccine priming has a significant impact on the mucosal IgA antibody response. Without inactivated vaccine priming, intranasal Ad5-S-Omicron only increased IgA and neutralizing antibodies against Omicron in BALFs and sera but not pre-Omicron strains Wildtype, Beta, and Delta. With inactivated vaccine priming, intranasal Ad5-S-Omicron elevated IgA and neutralizing antibodies against not only Omicron but also pre-Omicron strains in BALFs. This suggested that inactivated vaccine-primed memory B cells that cross-react with Omicron spike may further proliferate after intranasal Ad5-S-Omicron boost and home in the respiratory tract. Therefore, intranasal Ad5-S-Omicron on the basis of inactivated ancestral vaccine led to induction of broadly mucosal and systemic neutralizing antibodies. Of note, we observed a somewhat lower level of BALF IgA for Omicron BA.1 spike than for Wildtype spike, whereas neutralizing titer against BA.1 was higher than that against the Wildtype spike. This is probably due to the presence of common non-neutralizing epitopes among different spike proteins or the presence of more IgA-binding epitopes in the Wildtype spike. Intranasal booster using Ad5-S-Omicron not only stimulated new B cells producing Omicron-specific antibodies but also proliferated memory B cells that cross-reacted with both Wildtype spike and Omicron spike. Another possible reason is that different spike proteins used for ELISA were prepared at different times by the vendor, so the variations in quality may be attributed to this difference.

An ideal vaccine should be highly effective in blocking infection, easy to use, and well-accepted by the elderly and children. Although aerosolized adenovirus vector-based ancestral vaccines have been reported to be well tolerated and could induce good systemic neutralizing antibody responses in humans, thus far, the induction of spike-specific IgA in nasal tract after aerosolized vaccination has not been well reported.^24^ Another group reported that a lower level of spike-specific IgA in nasal mucosal samples could be detected in 4/13 participants after intranasal vaccination with a chimpanzee adenovirus ChAdOx1vectored ancestral vaccine AZD1222.^25^ As discussed in their report, adenovirus vectors derived from different species may lead to different immune responses in humans. The ChAdOx1 vector, which is derived from a simian adenovirus serotype, may have poorer infectivity than human adenoviruses to human nasal epithelium, resulting in low expression levels of spike antigen and thus lower immune response. In our study, we employed an Ad5 vector, which was originally isolated from human nasopharyngeal tract and can effectively infect human epithelial cells in the upper respiratory tract. Five co-authors of this report, who previously received 2 intramuscular injections of inactivated ancestral vaccine over 6 months ago, self-administered intranasal Ad5-S-Omicron, and all observed a significant induction of nasal secretory IgA. A previous study also reported that the human Ad5 vector conferred better immunogenicity than chimpanzee adenovirus vector ChAdOx1 in mice.^26^ However, the underlying mechanism is worthy of further investigation.

Most intriguingly, passive transfer of the nasal lavage fluid into mice could confer effective protection against Omicron challenge. This proof-of-principle experiment demonstrated that intranasal vaccination can establish a respiratory immunity wall to provide potent protection against Omicron infection. This observation also concurred with our previous report that intranasal vaccination with Ad5-S-WT (Ad5-S-nb2) conferred sterilizing immunity-like protection in macaques challenged with the SARS-CoV-2 Wildtype strain.^16^ Other studies also reported that intranasal vaccination using chimpanzee adenovirus vectored vaccines protects against SARS-CoV-2 infection through respiratory tract in animal models.^27,28^ Previous clinical trials of intranasal adenovirus vectored vaccines such as influenza have also demonstrated the detection of antigen-specific IgA in humans.^14^ Our study has great implications for controlling the infection and transmission of globally circulating Omicron variants. Several studies have reported that Omicron variants prefer to infect the upper airway, especially the nasal tract; therefore, a vaccine that can elicit a high level of neutralizing IgA in the upper respiratory tract could offer the first-line defense against infection and transmission of Omicron subvariants.^19^ It has been reported that people with a higher level of Wildtype spike-specific IgA in nasal swabs have a lower Omicron breakthrough infection and lower viral load.^22^ It appears that even low cross-reactivity against Omicron may still confer certain protection against Omicron infection.^22^ Indeed, mucosal dimeric IgA is approximately 15-fold more potent against SARS-CoV-2 than monomeric IgG.^29^ It has been suggested that secretory IgA may be more broadly cross-reactive against variants.^30^ Conceivably, an intranasal Omicron vaccine could be more effective than an ancestral vaccine in inducing Omicron-specific mucosal and systemic immunity.

In summary, intranasal Ad5-S-Omicron booster on the basis of an ancestral vaccine provides additional layers of protection compared to intramuscular vaccines, including mucosal IgA and residential T cells in the respiratory tract, in addition to systemic IgG antibody and T cell responses. Using Ad5-S-Omicron as an intranasal booster in people who previously received ancestral vaccines should be tested in future clinical trials, which may provide an option to stop infection and transmission of SARS-CoV-2 variants.

## Material and methods

### Adenovirus-vectored vaccine and Western blot analyses

The replication-incompetent recombinant adenovirus Ad5-S-Omicron was generated as Ad5-S-WT (Ad5-S-nb2) reported previously.^16^ In brief, the codon-optimized sequence named S-Omicron encoding SARS-CoV-2 S-Omicron BA.1 protein (EPI_ISL_6640919) was synthesized (Genscript, China) and amplified by PCR. The gene was inserted into the shuttle plasmid pGA1 and then recombined with pAd5ΔE1ΔE3 backbone in *E. coli* competent cells to construct pAd5-S-Omicron, which was transfected into HEK293 cells to rescue Ad5-S-Omicron. Ad5-S-Omicron was propagated, purified, and titrated. Ad5-S-WT (Ad5-S-nb2) is a replication-incompetent recombinant adenovirus that was previously reported.^16^

HEK293 cells were infected with Ad5-empty or Ad5-S-Omicron in 6-well plates. The cell lysates were harvested 24 h after infection, and SDS‒PAGE was performed. The PVDF membranes were incubated with SARS-CoV-2 S protein-specific rabbit monoclonal antibody. Then, PVDF membranes were incubated with secondary antibody (HRP-conjugated goat anti-rabbit antibody). PVDF membranes were incubated using a chemiluminescent HRP substrate and imaged by a Bio-Rad imaging system.

### Animals, viruses, and cells

Female 7-week-old BALB/c and hACE2 transgenic mice were supplied by Beijing Vital River Laboratory Animal Technology Co. Ltd. The mouse experiments were performed in the Animal Experimental Center of GIBH, CAS (IACUC: 2020025), and all protocols were approved by the Institutional Animal Care and Use Committees of GIBH. Omicron BA.1.1 and BA.2.3 were isolated from COVID-19 patients and preserved in Biosafety Level 3 (BSL-3) Laboratory of Guangzhou Customs District Technology Center. Works related to authentic SARS-CoV-2 virus were performed in BSL-3 Laboratory of Guangzhou Customs District Technology Center. This virus was passaged and titrated in Vero E6 cells. All experiments related to infectious SARS-CoV-2 were conducted using appropriate positive-pressure air respirators and protective equipment in BSL-3 and ABSL-3 facilities approved by the Institutional Biosafety Committee. HEK 293 cells (ATCC, CRL-1573), Vero E6 cells (ATCC, CRL-1586), A549 cells (ATCC, CCL-185), and Huh-7 cells (National Collection of Authenticated Cell Cultures, TCHu182) were cultured at 37 °C in 5% CO_2_ in complete DMEM supplemented with 10% fetal bovine serum (FBS), 100 U/ml penicillin, and 100 U/ml streptomycin.

### Collection of bronchoalveolar lavage fluids (BALFs) in mice

Mice were sacrificed by the cervical dislocation method after blood collection and dampened with 75% ethanol. The mice were secured, and the lungs were washed 3 times using 0.8 ml PBS with 2% FBS by 23-gauge lavage tube passage into the trachea twice. The retained BALFs were centrifuged at 400 × g for 5 min at 4 °C. The BALF supernatants were used for antibody detection, and the BALF cells were used for ELISpot assays.

### ELISA for spike-specific IgA

96-well plates coated with 0.05 μg S protein (Sino Biological Inc., China) were blocked with 1 × PBS containing 0.05% Tween-20 and 5% skim milk for 2 h at 37 °C. Serially diluted serum and BALF samples were added to 96-well plates and incubated for 2 h at 37 °C. The plates were incubated using HRP-conjugated goat anti-human IgA antibodies (Beijing ZSGB Biotechnology, China) for 1 h at 37 °C. 3,3′,5,5′-Tetramethylbenzidine (TMB) substrates were added and measured at 450 nm. The cutoff value was defined as 2.1-fold of OD450 values from the sample of nonvaccinated mice. The endpoint binding antibody titers were defined as the reciprocal of the maximum sample dilution with OD450 values equal to or greater than the cutoff value.

### IFN-γ ELISpot assay

Freshly isolated mouse spleen cells or BALF cells were subjected to ELISpot assays. In brief, 2 × 10^5^ splenic lymphocytes or 2.5 × 10^4^ BALF cells per well were added to 96-well plates (Merck Millipore) coated with mouse anti-IFN-γ antibody (R&D Systems) and then incubated with S peptide pools corresponding to Wildtype, Delta or Omicron BA.1 (Genscript, China) at 2 μg/ml per peptide for 24 h. Biotinylated detection antibodies and alkaline phosphatase-conjugated streptavidin (BD PharMingen) were added to the plates, and spots were developed with NBT/BCIP substrates (Pierce). Finally, spots in each well were counted by using an ELISpot reader (S7DX, CTL).

### Immunofluorescence analysis

A549 cells were incubated with Ad5-empty or Ad5-S-Omicron for 24 h. Treated A549 cells were fixed using 4% paraformaldehyde and blocked using 1 × PBS supplemented with 5% BSA. After labeling with S protein-specific rabbit monoclonal antibody, A549 cells were stained with FITC-conjugated goat anti-rabbit antibody. Finally, A549 cells were observed under fluorescence microscopy.

### Intranasal vaccination and collection of nasal lavage fluids (NLFs) in volunteers

The intranasal vaccination for humans was performed following the manufacturer’s instructions. In brief, 0.2 ml of vaccine (containing 2 × 10^10^ vp Ad5-S-Omicron) was aspirated with a syringe, and the syringe was then attached to a nasal spray device. The device was placed with the tip snugly against the volunteer’s nostril and compressed briskly to deliver half of the vaccine dose into the nostril. The procedure is repeated with the other nasal. Nasal lavage fluids were collected using a nasal wash irrigator with 40 ml saline and used for detecting IgA by ELISA. The 40 ml nasal lavage fluid was concentrated 20-fold using an ultracentrifugal filter with a 100 kDa molecular weight cutoff (Merck Millipore) for SDS‒PAGE, Western blot analyses, pseudovirus neutralization assays, and mouse adoptive transfer experiments.

### Mouse vaccination and challenge

For intramuscular vaccination, mice were injected intramuscularly with 100 µl adenovirus vectored vaccine or 50 µl inactivated vaccine into the muscle of the hind limbs (equally injected into the left and right hind limbs). For intranasal vaccination, mice were anesthetized by isoflurane, and adenovirus vectored vaccines were intranasally inoculated with appropriate viral particles (vp) in 50 μl PBS. Serum samples were collected at different times after vaccination. The mouse model of SARS-CoV-2 infection was generated and described previously.^31^ The vaccinated or adoptively transferred nasal wash mice were challenged intranasally with the specified SARS-CoV-2 Omicron BA.1.1 or BA.2.3. Mice were sacrificed and lungs were collected for virological immunological study 3 days post-challenge. All experiments related to SARS-CoV-2 virus were conducted in BSL-3 and ABSL-3 Laboratories of Guangzhou Customs District Technology Center.

### Multiplex electrochemiluminescence assays

Mucosal SARS-CoV-2-specific IgA was performed by a V-PLEX SARS-CoV-2 Panel 25 (IgA) Kit (K15585U, Meso Scale Diagnostics, Maryland, USA) according to the manufacturer´s instructions. Nasal lavage fluids were prediluted 1:12.5-fold before detection.

### Neutralization assay based on pseudotyped VSV

Pseudovirus neutralization assays were performed based on a previous study.^18^ Briefly, 3-fold serial dilutions of serum or BALFs or NLFs with a starting dilution of 1:30 (serum) or 1:10 (BALFs and NLFs) were coincubated with 800 TCID50 pseudovirus supernatants in a 96-well microplate (JETBIOFIL, China) for 1 h at 37 °C. Afterward, 100 μl of the mixture from each well was transferred to a white 96-well cell culture plate (Corning, USA), and 20,000 Huh-7 cells per well were added in turn. Following a 24-hour incubation at 37 °C and 5% CO_2_, the relative luminescence unit (RLU) reduction compared with virus-infected untreated control cells was evaluated by the Bio-LiteTM Luciferase Assay System (Vazyme, China) following the manufacturer’s instructions. The 50% inhibitory concentration (IC50) values for neutralization were calculated by the Reed-Muench method.

### qRT‒PCR

SARS-CoV-2 RNA quantity in the lung tissue was determined by one-step real-time quantitative RT‒PCR. The plasmid DNA carrying the original Omicron BA.1 S gene was used as a template. Lung tissue homogenate RNA was extracted and eluted using elution buffer (QIAamp Viral RNA Mini Kit, Qiagen). The primer set included forward primer (qF: 5′- CAGCAACTGTTTGTGGACCTA–3′) and reverse primer (qR: 5′- CTCAAGTGTCTGTGGATCACG–3′). Viral RNA copies were quantified according to the manufacturer’s instructions (HiScript II One Step qRT‒PCR SYBR Green Kit, Vazyme). The amplification procedures of quantitative RT‒PCR were as follows: 50 °C for 30 min, 95 °C for 30 s followed by 40 cycles containing 95 °C for 10 s, 50 °C for 30 s, and a default melting curve step (ABI QuantStudio 3 Real-Time PCR System). The standard curve was obtained based on serial dilutions of SARS-CoV-2 RNA fragments generated by in vitro transcription. The limit of detection (LOD) was determined by the standard curve and the dilution, which was approximately 1000 copies/g of biopsy samples. The viral loads were converted to the genome copies of SARS-CoV-2 in one g of tissue.

### SDS‒PAGE and Western blot analyses of nasal IgA

NLFs from donors were subjected to SDS‒PAGE and stained with Coomassie blue for SDS‒PAGE analysis. For western blot analyses, NLFs subjected to SDS‒PAGE were transferred from the gel to the PVDF membrane. The PVDF membranes were incubated with goat anti-human IgA antibodies (HRP-conjugated). PVDF membranes were incubated using a chemiluminescent HRP substrate and imaged by a Bio-Rad imaging system.

### Spleen lymphocytes

Mouse spleens were harvested and grinded with the flat end of a syringe in 8 ml of mouse lymphocyte-separating fluid on a 60 mm culture dish. The grinded mixture was filtered through a 200-mesh stainless mesh. The cell suspension was resuspended in 1 ml of DMEM and centrifuged at 800 × g for 30 min. The splenocytes were harvested and resuspended in 10 ml of 1640 medium, centrifuged at 250 g for 10 min, and resuspended in 3–5 ml serum-free medium.

### Statistical analyses

All quantifications were performed in a blinded manner. GraphPad Prism version 8.0 (GraphPad Software) was used for analysis of virologic and immunologic data. Unpaired Student’s t tests (two-tailed) were used for comparisons between groups. When *p* values were less than 0.05, the differences were considered statistically significant.

## Supplementary information

Supplementary Materials

## Data Availability

All experimental data generated or analyzed during this study are available from the corresponding authors upon reasonable request.
