# Supplementary material for: Intranasal booster using an Omicron vaccine confers broad mucosal and systemic immunity against SARS-CoV-2 variants
Source: Signal Transduct Target Ther. 2023 Apr 17;8:167. doi: 10.1038/s41392-023-01423-6 (PMC10106878; doi:10.1038/s41392-023-01423-6)
Supplement: Supplementary file 1 — Supplementary Materials [file 41392_2023_1423_MOESM1_ESM.docx]

Supplementary Materials for

**Intranasal booster using an Omicron vaccine confers broad mucosal and systemic immunity against SARS-CoV-2 variants**

Qian Wang^a#^, Chenchen Yang^c,d#^, Li Yin^b,e#^, Jing Sun^a#^, Wei Wang^d#^, Hengchun Li^b,e^, Zhengyuan Zhang^b,e^, Si Chen^d^, Bo Liu^c,d^, Zijian Liu^b,e^, Linjing Shi^b,e^, Xiaolin Liu^c,d^, Suhua Guan^c,d^, Chunhua Wang^c,d^, Linbing Qu^b^, Ying Feng^d^, Xuefeng Niu^a^, Liqiang Feng^b^, Jincun Zhao^a,d*^, Pingchao Li^b*^, Ling Chen^a,b,d*^, Nanshan Zhong^a,d^

^a^State Key Laboratory of Respiratory Disease, Guangzhou Institute of Respiratory Health, the First Affiliated Hospital of Guangzhou Medical University, Guangzhou, China

^b^Guangdong Laboratory of Computational Biomedicine*,* Guangzhou Institutes of Biomedicine and Health, Chinese Academy of Sciences, Guangzhou, China

^c^Guangzhou nBiomed Ltd.

^d^Guangzhou Laboratory & Bioland Laboratory, Guangzhou, China

^e^University of Chinese Academy of Sciences, Beijing, China

^#^These authors contributed equally

*Correspondence to: Ling Chen (chen_ling@gibh.ac.cn); Pingchao Li (li_pingchao@gibh.ac.cn); Jincun Zhao (zhaojincun@gird.cn);

**This PDF file includes:**

Figures. S1


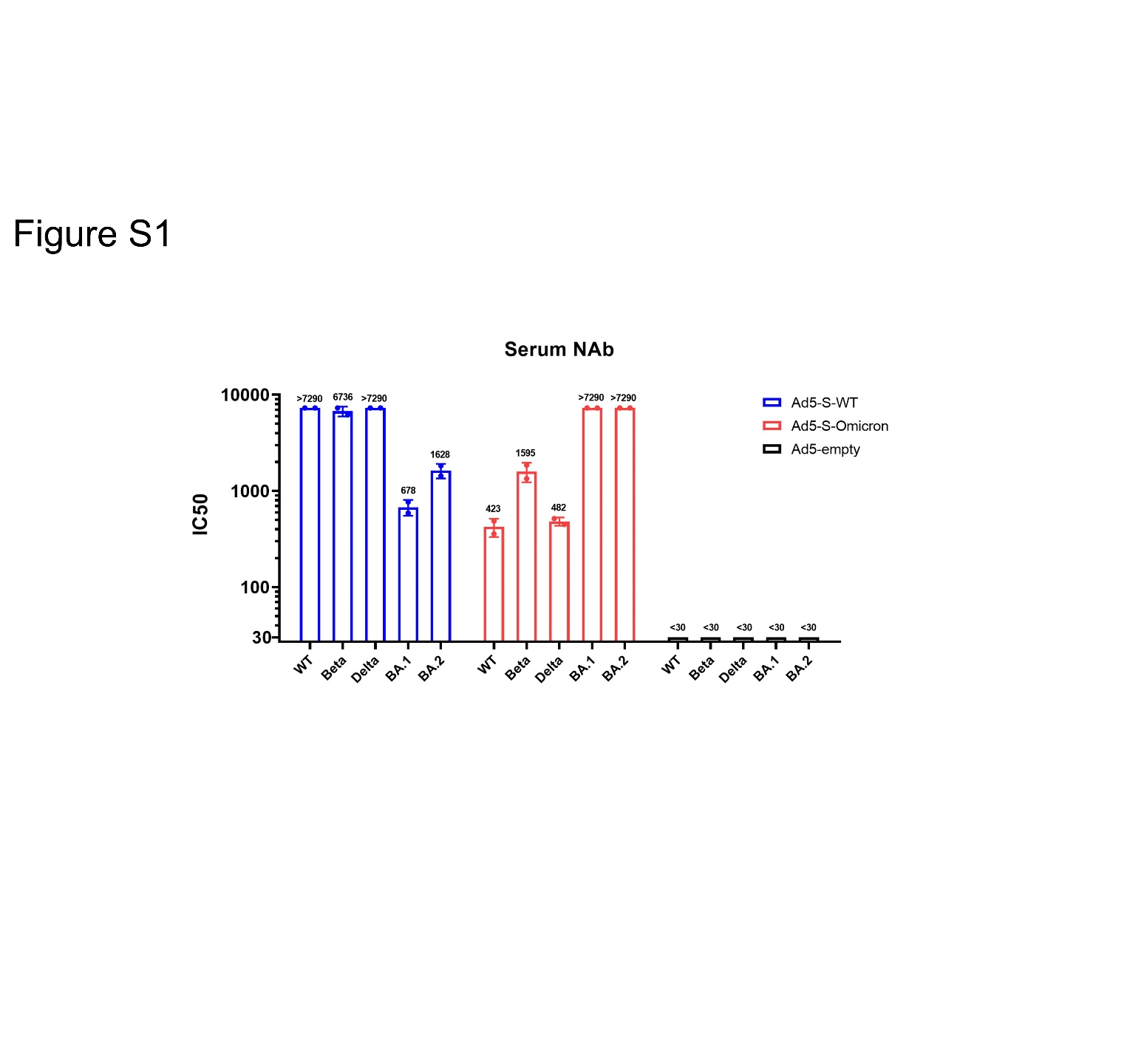


**Figure. S1. Comparison of neutralizing titers in mice that received either intranasal Ad5-S-Omicron or intranasal Ad5-S-WT.** Female 7-week-old BALB/c mice received a single dose of either intranasal Ad5-S-Omicron (2×10^9^ vp), intranasal Ad5-S-WT (2×10^9^ vp), or intranasal Ad5-empty (2×10^9^vp). At 6 weeks after vaccination, serum samples from each group of 5 mice were pooled in equal volume for detection of pseudovirus NAb titers against Wildtype, Beta, Delta, and Omicron BA.1 and BA.2 in duplicate. Mice that received Ad5-empty were used as controls and showed no neutralizing titers over 1:10.
